# Practical Notes

**Published:** 1854-10

**Authors:** 


*Practical Notes.— Chloroform.*—Dr. G. W. Ronald, in the Western Journal of Medicine and Surgery, details a case of rigidity of the os uteri during labor, which had resisted the lancet, tartrate of antimony, and the warm bath; which yielded very readily to the application of the chloroform ointment to the os. He has used this ointment in but a small number of cases, but thus far successfully. One drachm and a-half of chloroform is thoroughly incorporated with one ounce of simple cerate. This may answer very well as a local application in places where the chloroform liniment cannot be conveniently applied. The chloroform liniment is one of the neatest and most active of external means in relieving pain. The usual formula is

℞ Chloroform,
Gum camphor,
Olive oil, $\overline{aa}$ ℥j. M.

We find in the New York Journal of Medicine an extract from an English journal, in which chloroform was given by inhalation, in the excessive restlessness which sometimes follows uterine, or other hæmorrhage. The patient had lost much blood, and was in a sinking condition; the pulse almost imperceptible; the breathing distressed and gasping; and there was great exhaustion, nervous excitement, jactitation, and desire for sleep. Brandy was given freely, with fifty drops of laudanum, followed by seventy drops more in a quarter of an hour. Seeing that repose was indispensable, he administered chloroform, when the patient came immediately under the influence of the opium previously taken, and slept for two hours, awaking much refreshed. Some doubts may arise as to the propriety of giving 120 drops of laudanum in fifteen minutes, to a feeble woman suffering under great nervous exhaustion. That so sedative a dose was calculated to increase the agitation is more than probable. Again, it required some heroism to give chloroform to anæsthesia under these circumstances, as it is known that opium, followed by chloroform, has a sudden cumulative effect which is sometimes dangerous. Alarming coma has occurred in this way.

The case is, however, instructive, and occasion may arise for the adoption of this treatment. Very recently we treated a case of delirium tremens, in which, having given a grain of sulph. morph. hourly, for four hours, with brandy, the patient grew alarmingly excited and frantic. We then administered chloroform by inhalation, until sleep was produced, which occurred in a very little while. The patient slept then for five hours. Soon after waking he became again somewhat excited, and again inhaled the anæsthetic,

After a sleep of several hours, he awoke calm, and went on to recovery; bu immediately on getting about renewed his habit of drinking, and, in the delirium it soon occasioned, jumped from a third story window, breaking the tibia. He was removed to the hospital, and, as is common in such cases, died of the combined effects of disease and the accident; thus terminating a life marked by vicissitude and crime.

In very many cases where opiates have been given without producing sleep, the inhalation of a little chloroform will at once bring the system under the influence of the narcotic. Proper caution is necessary that its effect be not too deep or sudden.

*Counter-poisons.*—Dr. Graves, (of Dublin, we presume,) having suggested that belladonna was indicated in the coma of continued fever *with contracted pupil*, Dr. Thomas Anderson, of Edinburgh, tried the experiment of attempting to remove the poisonous effects of opium by belladonna, arguing that if belladonna would remove the contracted pupil of the opium coma, it might also, remove the other comatose symptoms. Accordingly, to a man who had taken a large overdose of morphia, he gave the following mixture:

℞ Tinct. belladonna, ʒvi,

Aqua, ℥vss. M.

Take an ounce every half hour.

This was continued until dilatation of the pupil came on. The effect upon the brain was such as was anticipated. The coma disappeared, the pulse returned, and the patient went on well for three days, when, on suddenly assuming the erect position, syncope occurred, and death.

Nothing discouraged, Dr. Anderson made use of the remedy in a second case, after the usual remedies, (motion, the stomach pump, and the galvanic current,) had failed to make any impression. The same dose was given every half hour, beginning five hours after the laudanum was taken. Three drachms of the tinct. belladonna were given in the course of an hour. In an hour and a half the pupil began to dilate, and with it the other symptoms to improve, until three hours afterward, when she was entirely free from the effects of the opium, and talked coherently. She recovered rapidly.

These cases are interesting, as indicating a more careful analysis of the effect of remedies, and the philosophy of their symptoms, than we often witness. Here one narcotic poison is given to remove the effects of another, upon a reasoning purely *a priori*, from a single symptom, viz., the effect upon the pupil.

The number of cases is insufficient to establish the practice. A committee, appointed by the Medical Society of Edinburgh to prosecute the investigation upon the lower animals, reported that the effects of these remedies upon them was so different from their effect upon the human system, that they were unable to arrive at any conclusion.

*Iodine.*—Of all the new remedies which the last twenty years have introduced to the profession, no one has gained so sure and useful a popularity as iodine. It is especially in its external uses that we now allude to it. In the form of tincture, its use may with propriety supersede many of the more usual counter-irritants. In strumous, or chronic peritonitis, it constitutes one of the most useful counter-irritants, both with relation to its revulsive effect, and the advantage to be gained by its absorption into the system. The tincture or alcoholic solution is the neatest and most convenient form of use. The ointment is liable to the objection that it greases and stains the garments. The tincture, when applied with a brush, dries so rapidly that it does not discolor the garments.

In all wounds by animal poisons, it is probably the most efficacious of remedies. Cases of rattlesnake bites have been rendered innocuous by its use. We have reason to believe that it once saved us from the consequences of a dissection-wound. The part punctured becoming painful during the night, we applied it freely, and, as that was the last of the symptoms, it is probable that the iodine had an agency in prevention. In no accident is its efficiency more beautifully illustrated than in the sting of bees. A little child, lying in its cradle, was attacked by a swarm of bees in one of their annual *emeutes*, and was badly stung in numerous places, particularly upon the eyelids. The stings occasioned much distress and swelling, and the accident was really a serious one. We painted the swollen spots with the alcoholic solution; the relief was almost immediate, and the swelling subsided so rapidly, that in a few hours hardly a trace of the accident remained. Applied to a bee sting its action is abortive, and from analogy, as well as from the published reports of cases, there is every reason to suppose that it will prove correspondingly beneficial in the more malignant animal poisons.

There is also an ethereal solution of iodine which is occasionally useful in producing an eschar. The affinities of ether for iodine, are very strong, and the ethereal solution is a very active escharotic. A bit of cotton moistened with it applied to the skin, and covered with a wet towel to prevent evaporation, will act as a caustic in fifteen or twenty minutes, producing a superficial sore. Its action is accompanied by severe pain.

*Salt as a Preservative of Dissecting Material.*—About the first of December, 1853, a fine muscular subject was injected through the femoral artery with a solution containing 3lbs. of nitrate of potash. It was then packed in a solution of common salt. It was dissected during April, and found to be in excellent preservation; the muscles being unusually red, a quality, to obtain which, the injection was used. It kept better on the table than any other subject we ever saw, lying for a month of warm weather without much decay, the muscles drying down instead of softening by putrefaction.

Another subject was packed in the brine about the 1st of January, the injection being omitted. This subject has stood during the entire summer in a room lighted by a large window, with a southern exposure, the barrel in which it was placed being open to the air at the top. After nearly nine months of this hard usage it is now upon the table in capital preservation. The advantages of this mode of preservation are these:

1st. It is cheap and certain.

2d. The tissues are dryer than when preserved in the usual manner, and the dissection consequently cleaner and less obscure.

3d. The nitrate of potash gives a lively red to the muscles.

4th. The subject bears exposure upon the table better, and does not communicate that extremely offensive smell to the hands, which accompanies ordinary dissections. A careful washing removes all smell.

All good things have their drawbacks. The fault in these subjects is a hardening of the fingers and toes, which renders dissection of those parts well nigh impossible, as they cannot be softened by water to any extent. This hardening is attributed to the presence of an excess of salt in the solution. If this supposition should prove to be correct, we can conceive of no better means of preserving *material* for months, or years, than by common brine, as subjects thus preserved are (with the exception we have mentioned) really more pleasant to dissect than a fresh subject.

*Glandular Enlargement after Dysentery and Cholera.*—The attention of some members of the profession has been recently called to a sequela of cholera and dysentery, consisting of a swelling in the neck, commencing usually in the parotid gland, or possibly in the cellular tissue over it, which gradually extends, involving the other glandular structures of the neck, and finally causing death by pressure upon the larynx and bloodvessels. The swelling has the same hard, "crockery" feeling beneath the skin, which is sometimes noticed under the chins of strumous children. When it covers several glands it is lobulated, the skin over it is of a dusky red color, without much tenderness, or excitement of the circulation. In the only instance occurring in our own practice, it appeared during convalescence, and this, we believe, holds true in other cases. It has been suggested by a medical friend, that it only happened in cases where the acetate of lead had been previously exhibited. This is an important point to settle, as it would in some degree inhibit the use of that drug. Since this suggestion has been made, two cases following cholera have occurred in this city, in neither of which had sugar of lead been given. One of these cases suppurated in the region of the parotid, and after a tedious illness the patient recovered. The other terminated in resolution, the ung. iodid. being freely applied. The case in our own practice recovered by suppuration. In the two other cases of which we have knowledge, neither showed any tendency to suppuration, and the swelling extended steadily toward the front of the neck, until it terminated the life of the patients by asphyxia. In both of these the lead had been given.

It is possible that this description includes two distinct diseases: one indolent and progressing to induration; the other acute and inclined to suppuration. But their apparent alliance with disease of the bowel indicates that they are rather two forms of the same action.

We are not aware that this is anywhere mentioned as one of the sequelæ of dysentery, or cholera. But it is evident from the number of cases already collected, that they stand in the relation of cause to the swelling; and that it may constitute a very serious complication, and one almost necessarily fatal in the indurated variety.

Any of our readers who may have witnessed swelling or abscess of the cervical glands following bowel complaints, would confer a material favor by a description of the cases. The relation, (if any it has,) of sugar of lead to the causation should also be considered.

---

*The Modern Treatment of Syphilitic Diseases, both Primary and Secondary. comprising the Treatment of Constitutional and Confirmed Syphilis, by a Safe and Successful Method.* By LANGTON PARKER, Surgeon to the Queen's Hospital, Birmingham. From the third, and entirely rewritten, London edition. Philadelphia: Blanchard and Lea.

The implied boast on the title-page of this volume is not calculated to prepossess the critic in its favor. A more careful examination, however, proves it to be a book decidedly English in its character; that is, in no place profound or brilliant, and sometimes offensive, from assumption of superior skill. It is still highly practical, and generally judicious in its comments.

The tedious discussions, which have marked the more recent works of Ricord, are absent; the pathology and theory of the disease are but cursorily considered; while the great bulk of the volume is devoted to the discussion of the different varieties of treatment. In this the author takes sides somewhat warmly with the mercurialists: looking upon mercury, in some of its preparations, as the sheet anchor of all successful treatment. He lays great stress upon mercurial fumigations, and attributes all the secondary results, which have been charged to calomel, to the improper and unscientific use of that drug. To those who already possess the works of Ricord and of Vidal du Cassis, this volume will not come amiss; presenting, as it does, the more practical and empirical view of the disease in question.

For sale by Miller, Orton & Mulligan. H.

---

*Healthy Skin: a Popular Treatise on the Skin and Hair, their Preservation and Management.* By Erasmus Wilson, F. R. S. Second American, from the fourth and revised London edition. Philadelphia: Blanchard & Lea.

This is a reprint of a small volume which appeared from the same press, with a slightly different title, several years ago.

It is a very clever popular treatise; not such an one as the practitioner would rely on for cases of difficult diagnosis, or to which he would refer for instructions in treatment to any extent. But for the popular reader, it would answer as well as any popular medical work, inasmuch as the patient is usually directed, at the close of each section or chapter, to apply to his physician.

Diseases of the skin are perhaps more difficult to explain to the common mind than those of any other organ. Their study is encumbered with a nomenclature and classification as intricate and unpronounceable as the older botanic systems. Added to this, it requires a nice eye for form and color to recognize the shades of difference which exist, and which are sometimes very important in treatment. Such difficulties indicate that skin diseases are a specialty, to be thoroughly understood only by those who make them a special subject of study.

For sale by Miller, Orton & Mulligan. H.

*The Pathology and Treatment of Pulmonary Tuberculosis; and on the Local Medication of Pharyngeal and Laryngeal Diseases frequently mistaken for, or associated with, Phthisis.* By JOHN HUGHES BENNETT, M. D., F. R. S. E. Philadelphia: Blanchard & Lea. 1854.

Some time last winter we received a copy of the Edinburgh edition of this admirable treatise, from its author. Our March number (if we recollect rightly) contained a somewhat lengthy review, or rather *resume*, of its contents.

It is unnecessary for us to add anything to the opinion then expressed. Messrs. Blanchard and Lea have republished it in a neat and cheap form. We hope that it may have, what it most richly deserves, an extended sale.

For sale by MILLER, ORTON, & MULLIGAN. H.

---

*A Clinical Introduction to the Practice of Auscultation, and other modes of Physical Diagnosis, in Diseases of the Lungs and Heart.* By H. M. HUGHES, M. D., F. R. C. P., Assistant Physician to Guy's Hospital. Second and revised edition. Philadelphia: Blanchard & Lea. 1854.

This little work is intended as an elementary treatise on physical diagnosis, more especially for the use of students. The style is accordingly direct and simple; the instructions clear; and the subject divested of any undue mystery. There is an unusual freedom from technical terms, and a familiarity in its manner, which makes it a very readable book. Without any pretensions to exhausting the subject, it is all that it claims to be, and a little more: that is, it forms a convenient book of reference to the practitioner upon occasion.

For sale by MILLER, ORTON & MULLIGAN. H.

---

*Ranking's Half-Yearly Abstract of the Medical Sciences* is received. It makes quite a respectable volume in itself. Such a collection of the new facts, reasonings, theories, and experiences of medical science, condensed into a single volume twice a year, makes a very valuable addition to one's library.

The method of its collection looks a little like piracy; but we do not find that any writer objects to it, and the really useful character of the thing when complete will atone for its free and easy appropriation of other men's thoughts. In any other branch of literature it would be insufferable; but in medicine, copy-rights to magazine papers are not very valuable, and pecuniary interests are so small as to be uncared for. Then, too, the reputation of the individual writer—and that is the thing usually written for—is largely benefited by a circulation of his literary wares in the pages of Rankin or Braithwaite.

Buy one or the other by all means. H.

---

*Malpractice Suit.*—At a recent session of the Circuit Court, in this city, the case of Adolphus Gates *vs.* John J. E. Haksteen, in an action for malpractice, resulted in a verdict for the plaintiff of $700, with costs.

We shall, in our next number, give some account of the circumstances and evidence in this trial. The result was unexpected by those who listened to the evidence, and is a very severe infliction upon the defendant, who is a reputable German practitioner in this city. H.

---

*Extra Session of the Erie County Medical Society.*—A call for an extra meeting, is circulating as we go to press, to take into consideration the recent violation of the society's fee bill for services to the county. The repeated outrages committed on the profession by the public authorities, render a prompt and vigorous action necessary to sustain the position we have lately assumed. Let the society preserve its own character and dignity, at whatever expense of personal feelings. H.

